# Diagnostic Implications of White Matter Tract Involvement by Intra-axial Brain Tumors

**DOI:** 10.7759/cureus.19355

**Published:** 2021-11-08

**Authors:** Saqib Kamran Bakhshi, Ayesha Quddusi, Shaikh D Mahmood, Muhammad Waqas, Muhammad Shahzad Shamim, Fatima Mubarak, Syed Ather Enam

**Affiliations:** 1 Neurosurgery, Aga Khan University Hospital, Karachi, PAK; 2 Medical College, Aga Khan University Hospital, Karachi, PAK; 3 Centre for Neuroscience Studies, Queen's University, Kingston, CAN; 4 Neurosurgery, University at Buffalo, State University of New York, Buffalo, USA; 5 Radiology, Aga Khan University Hospital, Karachi, PAK; 6 Surgery, Aga Khan University Hospital, Karachi, PAK

**Keywords:** white matter tracts, white matter changes on mri, intra-axial brain tumor, dti, tractography

## Abstract

Introduction

Diffusion tensor imaging (DTI) is being increasingly used during brain tumor surgery. However, there is limited data available on its diagnostic and prognostic value. Our objective was to assess the pattern of involvement of white matter tracts (WMTs) by intra-axial brain tumors on DTI. Secondary objectives were to evaluate implications of involvement of WMT on surgical resection, and the post-operative functional outcome.

Methods

This was a retrospective study of consecutive patients, who underwent DTI-guided surgery for brain tumors. The involvement of WMTs by tumors on DTI was assessed by a radiologist (who was blind to the pathology) using the Witwer classification. The pathology was reported by histopathologists using the World Health Organization brain tumor classification. Karnofsky Performance Status Scale (KPS) was used for assessing patients’ neurological status at admission, and at follow-up.

Results

Forty-five (58.4%) out of 77 tumors reviewed caused infiltration of WMTs, whereas only 22 (28.6%) tumors caused displacement of WMTs (p= 0.040). Among 32 cases of astrocytoma, the involvement of WMTs was influenced by the grade of tumor (p= 0.012), as high-grade tumors caused infiltration (19; 59.4%), unlike low-grade tumors that commonly caused displacement (2; 50%). Oligodendrogliomas caused infiltration/disruption of WMTs in most cases, irrespective of the grade (19 out of 25 cases; 76%). At the last follow-up, 27 (35.1%) patients showed improvement in KPS and 14 (18.2%) reported deterioration, while there was no change observed in 36 (46.8%) patients. The infiltration of WMTs was associated with a poor functional outcome.

Conclusions

High-grade astrocytomas mostly cause infiltration of WMTs, unlike oligodendrogliomas, which often infiltrate WMTs, irrespective of the tumor grade. The infiltration of WMTs is associated with a poor functional outcome at follow-ups.

## Introduction

Magnetic resonance imaging (MRI) is the standard radiological modality for brain tumor imaging, both before and after surgery [1,2]. While conventional MRI provides adequate clinically relevant information, and delineates between white and grey matter, it does not show axonal tracts that make up the white matter. Diffusion tensor imaging (DTI) is an imaging modality that represents white matter tracts (WMTs) and allows a clear, reliable visualization of their orientation [3,4]. Neurons, like all other cells, contain water molecules that are in constant random motion (Brownian motion). The random movement of molecules of any particular substance can either be uniform in all directions (isotropic), or it can be variable in different directions (anisotropic). Water molecules in the WMTs exhibit anisotropic motion as axonal membrane and myelin sheath restrict the movement of these water molecules in the direction of the axon [3]. DTI uses this information to produce detailed 3D images that demonstrate the orientation, microstructural integrity, and composition of WMTs [1-4].

DTI also provides us with information on the effect of any space-occupying lesion (SOL) on the architecture of these WMTs. This information is helpful in planning the surgical approach and excision of both neoplastic and non-neoplastic lesions [5-7]. Furthermore, the effect of a neoplastic lesion on WMTs as demonstrated by DTI can also be useful in predicting the grade of the neoplastic lesion based on imaging alone [8,9]. DTI can be elaborated using indices such as fractional anisotropy (FA), to further describe the relation of WMTs with SOLs [4,6,10,11].

We conducted a study to observe the effect of intra-axial brain tumors on WMTs, as demonstrated by DTI. We also aimed to review the utility of DTI for surgical planning, as well as predicting the grade of brain tumors.

## Materials and methods

This was a retrospective study of 77 consecutive patients who had pre-operative DTI scans, and subsequently underwent craniotomy and tractography guided excision of intra-axial brain tumors at the Aga Khan University Hospital (AKUH), Karachi, Pakistan, during 12 months. All brain tumor patients at our hospital undergo surgery using intra-operative neuro-navigation with an option for DTI. Every tumor case was discussed in a multidisciplinary team meeting involving dedicated medical oncologists, radiation oncologists, pathologists, neuro-radiologists and neurosurgeons. Approval for the study was obtained from the University Ethics Review Committee (4906-Sur-ERC-17). Data was collected from our medical records, using a pre-designed and pre-tested form, and included patient details such as demographics, clinical presentation, in-hospital management, extent of WMT involvement, histopathological diagnosis, and post-operative functional outcomes at the last follow-up after surgery. Patients with incomplete records and extra-axial lesions were excluded from the study. The WMTs that were observed included corticospinal tract, corticopontine tract, corticobulbar tract, anterior corona radiata, superior corona radiata, posterior corona radiata, superior thalamic radiation, anterior thalamic radiation, anterior and posterior limbs of internal capsule, superior longitudinal fasciculus, inferior longitudinal fasciculus, medial longitudinal fasciculus, dorsal longitudinal fasciculus, cingulum, corpus callosum, short association fibers, external capsule, uncinate fasciculus, forceps major, forceps minor, transverse pontine fibers, central tegmental fibers and fornix.

MRI and DTI were performed on a 1.5 Tesla machine, and MR images including T1, T2, fluid-attenuated inversion recovery (FLAIR), T1 post-contrast and FA maps were reviewed by a consultant neuro-radiologist who was blinded to the final histopathological diagnosis. We used the classification system proposed by Witwer et al. to classify the extent of involvement of WMTs by the brain tumors using degree of FA into unaffected (FA: 0.163-0.286), displaced (FA: 0.085-0.093), edematous (FA: 0.092-0.149), infiltrated (FA: 0.050-0.059) and disrupted (FA: <0.050) [12]. The tracts were said to be displaced when they maintained nearly normal anisotropy relative to the corresponding tract in the contralateral lobe, but were situated in an abnormal location, and edematous if they demonstrated hyperintensity on MR T2-weighted images while maintaining slightly irregular to normal anisotropy and orientation. In case the tumor caused reduction in the fractional anisotropy of identifiable tracts, they were labeled as infiltrated, whereas non-identifiable tracts with markedly reduced anisotropy were labelled as disrupted tracts.

Gross total resection (GTR) was defined as no radiological evidence of residual tumor on post-operative MRI, whereas maximum safe resection (MSR) was defined as the removal of tumor volume to such an extent where neurological function could be preserved. Biopsy was defined as the removal of only a small amount of tissue that was sufficient for histological analysis. The World Health Organization (WHO) 2016 classification was used to diagnose and grade tumors by the histopathologists of our hospital.

Karnofsky Performance Status Scale (KPS) was used to determine the pre-operative and last follow-up functional status of patients [13]. The post-operative follow-up at our hospital is usually 10 days after discharge, but later follow-ups are variable with no set schedule of visits.

Statistical analysis

Statistical analysis was done using Statistical Package for the Social Sciences version 22 (IBM Corp., Armonk, NY). Means and standard deviations were calculated for continuous data with normal distribution whereas median and interquartile range (IQR) were calculated for continuous data with skewed distribution. Percentages and proportions were calculated for categorical data. Chi-square test and Fisher's exact test were used to compare categorical variables for parametric and non-parametric data, respectively. A p-value less than 0.05 was taken as significant. Change in functional status after surgery, histopathology and extent of tumor resection were correlated with the extent of involvement of WMTs.

## Results

Seventy-seven patients were part of the study. The mean age was 40.7 ± 14.8 years, and the most common presenting complaint was headache (n = 33; 42.9%). The median duration of presenting symptoms was 60 days (IQR = 15.5-225 days). Frontal lobe was the most common site of lesions (n = 38; 49.4%). A little more than half of the patients underwent surgery under general anesthesia (n = 46; 59.7%); the rest underwent surgery under local anesthesia with scalp block and fully awake craniotomy protocol. Sixty-seven patients (87%) had their post-operative MRI scan done immediately after surgery. Patients’ demographics and clinical characteristics are shown in Table 1.

**Table 1:** Demographics and clinical characteristics IQR, interquartile range *The term is not included in the latest WHO classification; however, our data included older cases as well with this diagnosis. **Includes less common cases such as gliosarcoma, ganglioglioma, ependymoma and sub-ependymoma.

Variables	Results (patients = 77)
Age (mean)	40.7 ± 14.8 years
Gender	
Male	54 (70.1%)
Female	23 (29.9%)
Co-morbid conditions	
Hypertension	16 (20.8%)
Diabetes	12 (15.6%)
Length of stay in hospital (median)	5 days (IQR = 4-7 days)
Duration of presenting symptoms (median)	60 days (IQR = 15.5-225 days)
Symptoms	
Headache	33 (42.9%)
Seizures	30 (39%)
Hemiparesis	16 (20.8%)
Aphasia	7 (9.1%)
Drowsiness	2 (2.6%)
Location	
Frontal	38 (49.3%; left = 23, right = 15)
Parietal	7 (9.1%; left = 4, right = 3)
Temporal	7 (9.1%; left = 5, right = 2)
Occipital	1 (1.3%; left = 0, right = 1)
Multiple lobes	21 (27.3%; left = 7, right = 10; midline = 4)
Cerebellar	3 (3.9%; left = 1, right = 2)
Anesthesia	
General anesthesia	46 (59.7%)
Scalp block (awake)	31 (40.3%)
Extent of resection	
Gross total resection	30 (39%)
Maximum safe resection	34 (44.2%)
Biopsy	3 (3.9%)
Post-operative scan not available	10 (13%)
Histology	
Astrocytoma (all grades)	32 (41.6%)
Oligodendroglioma (all grades)	25 (32.5%)
Oligoastrocytoma* (all grades)	6 (7.8%)
Lymphoma	4 (5.2%)
Metastasis	4 (5.2%)
Others**	6 (7.8%)

Functional outcome

At the last follow-up in clinic after surgery (median time: 0.6 months; IQR: 0.23-4.0 months), 27 patients (35.1%) showed improvement in their performance status (KPS), 14 patients (18.2%) had deterioration and 36 (46.8%) showed no change. Among patients who underwent GTR, 8 had improvement in functional status, 6 had deterioration and 16 had no change (p = 0.163). All patients who had deterioration in their performance status had malignant tumors (p = 0.006). The relationship between the involvement of WMTs by tumors and post-operative functional outcome is shown in Table 2 (p = 0.127).

**Table 2:** Relationship between change in the functional outcome and white matter tract involvement (p = 0.127)

White matter tract involvement	Clinical outcome (Karnofsky Performance Status Scale)		
	Improvement	Deterioration	No change
Unaffected	1	1	0
Displacement	6	1	15
Edema	0	0	0
Infiltration	17	10	18
Disruption	3	2	3

Involvement of WMTs

Tumors caused displacement of WMTs in 22 (28.6%) cases, infiltration of the tracts in 45 (58.4%) cases and disruption in 8 (10.4%) cases (p = 0.040). Table 3 shows the involvement of WMTs by different tumors of different grades.

**Table 3:** Involvement of white matter tracts by tumors of varying histopathology

	Unaffected	Displaced	Edematous	Infiltrated	Disrupted
Astrocytoma I	1	1	0	0	1
Astrocytoma II	0	1	0	0	0
Astrocytoma III	0	2	0	0	0
Glioblastoma IV	0	7	0	17	2
Lymphoma	0	1	0	2	1
Metastasis	1	0	0	2	1
Oligoastrocytoma II	0	1	0	1	1
Oligoastrocytoma III	0	2	0	1	0
Oligodendroglioma II	0	2	0	10	1
Oligodendroglioma III	0	2	0	9	1
Others	0	3	0	3	0

**Figure 1:**
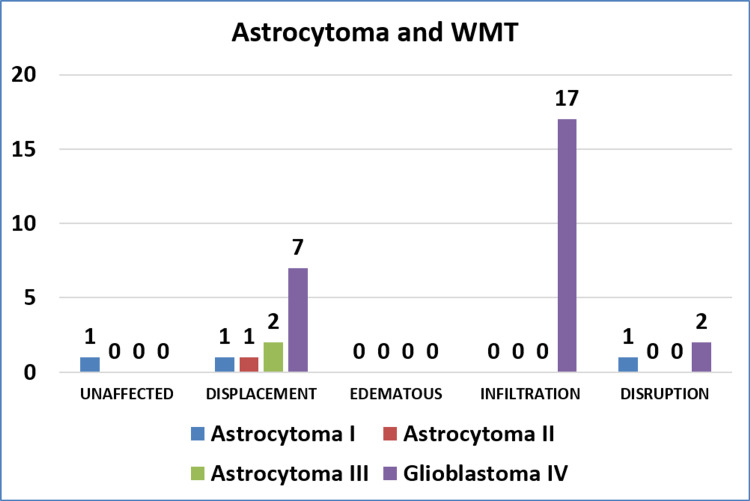
Involvement of WMTs by astrocytoma WMT, white matter tract

**Figure 2:**
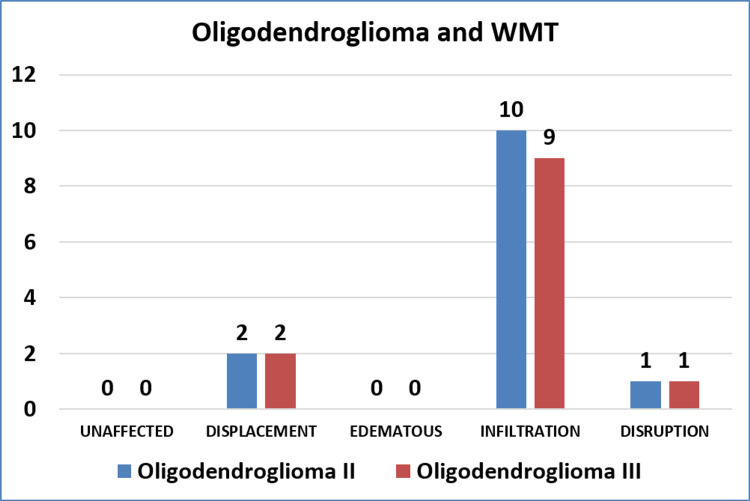
Involvement of WMTs by oligodendroglioma WMT, white matter tract

Among the 32 cases of astrocytomas, high-grade tumors (grade III and IV) more often caused infiltration of the tracts, and low-grade tumors caused displacement more often (p = 0.017). Contrary to this, among the 25 cases of oligodendrogliomas, the involvement of WMTs was not associated with the grade of tumor (p > 0.999). Figure 1 and Figure 2 show grade-wise distribution and relationship with WMTs, of astrocytoma and oligodendroglioma, respectively.

## Discussion

Surgery for brain tumors requires maximum resection of the lesion for better effect of chemotherapy and radiotherapy, as well as maximum preservation of fibers in functionally significant areas to avoid the deterioration of neurological status. In this context, DTI is useful in pre-surgical planning as it allows the surgeon to visualize viable WMTs on imaging. Several studies have reported good outcomes with the use of DTI [14-16]. In our experience, DTI had utility in pre-operative planning for surgical approach to avoid damage to vitals tracts, but it did not help much in improving the extent of resection or the functional outcomes, as discussed later.

At a median follow-up of three weeks from surgery, 14 patients (18%) had persistent deterioration in their neurological function (KPS) as compared to pre-operative examination. All 14 of these patients had malignant glial tumors on histopathology (p = 0.006). Out of these, 10 patients had infiltration of WMTs by their lesions but this association was not statistically significant with a p value of 0.127. The likely reason for this relationship not being statistically significant is the fact that out of 40 cases where tumor was infiltrating the tracts, subtotal resection was performed in more than half of the cases (n = 22; 55%). Most other studies that have assessed the role of DTI in tumor surgery report deterioration in neurological function in 12%-24% patients [17]. These are significant results, because based on these, DTI can be considered a tool to predict post-operative neurological deterioration, or the chance of neurological recovery.

The gold standard for tumor grading is histopathological examination of the biopsy of the lesion. Recently, molecular features have also been added to the grading along with the previously used histopathological characteristics [18]. Over the years, different imaging modalities such as diffusion-weighted imaging (DWI), and dynamic contrast-enhanced MRI have also been studied for their potential use in tumor grading [19,20]. More recently, great interest has been taken in studying high-grade and low-grade tumor characteristics using DTI. Several studies have looked at the relation of various DTI indices such as FA, apparent diffusion coefficient and others with relation to high-grade and low-grade lesions [21-28]. FA has been used to differentiate between high-grade and low-grade meningiomas. While some studies have found that FA is increased in high-grade meningiomas, others have found that it is decreased in high-grade meningiomas [21-25]. Similarly, DTI indices have been used to differentiate between high-grade and low-grade gliomas. In a prospective study of 35 patients, a combination of mean diffusivity (MD) and FA was considered reliable for differentiating between high-grade and low-grade gliomas [26]. Another study found the fiber density index (FDi) of low-grade gliomas was higher than that of high-grade gliomas [27]. FA and FDi ratios have also been observed to be different in high-grade versus low-grade gliomas [28]. We could only measure FA values in our cases, and used them as one of the ways to categorize tract involvement. However, it is a limitation of our study that we did not analyse and correlate individual values with regard to different parameters; we plan to assess this in future studies.

In our study of 77 patients with various types of intra-axial brain tumors who underwent pre- and post-operative DTI imaging, we found that tumors caused displacement of WMTs in 22 patients, infiltration in 45 patients and disruption in 8 patients. The involvement of WMTs was significantly associated with grade in astrocytoma (p = 0.012). However, on holistic analysis of the data, we did not find any statistically significant difference in the involvement of WMTs by different grades of tumors. WMTs were infiltrated by high-grade tumors in 60.4% cases, and by low-grade tumors in 54.2% cases. High-grade tumors caused displacement of WMTs in 28.3% cases, and low-grade tumors caused displacement in 29% cases. Khan et al. conducted a prospective study involving 128 patients who underwent pre-operative DTI and were subsequently operated for brain tumors [17]. They reported that 75% low-grade gliomas in their study population caused displacement of tracts and 71.25% high-grade gliomas caused infiltration/disruption of tracts. Similar findings were shared by Ibrahim et al. who conducted a smaller study comprising 32 patients and found that 90.9% low-grade gliomas displaced WMTs and 57.1% high-grade gliomas infiltrated/disrupted WMTs [29].

Our results show that intra-axial tumors were more likely to cause infiltration of WMTs, irrespective of the grade in most of the cases. Several other authors have explored these characteristics and some have suggested using this information along with other radiological features such as MR spectroscopy and MR perfusion, to arrive at a pre-operative diagnosis with certainty [30]. However, we could not support the results with specificity and sensitivity, because of the smaller sample size of tumor subtypes.

There are few notable limitations of this study including the absence of a control group for comparison (surgery without DTI), and a smaller sample size that did not allow us to perform advanced statistical analysis including multivariate regression analysis. Because of the retrospective design, we could not observe and analyse some parameters including the frequency of cases where resection was limited due to the presence of tracts and the association of tractography with cortical stimulation during awake craniotomy cases. However, considering the limited literature on this topic from the developing world, and the emerging nature of this technology, our results raise questions that will derive further research in this area. We recommend larger prospective studies to further assess the role of regular pre-operative MR DTI for intra-axial brain tumors in predicting the grade of the lesion and the risk of developing post-operative deficits. We also recommend using this technology to predict molecular features of histopathological diagnosis based on neuro-imaging, which will have a profound effect on pre-operative counselling, decision making and treatment planning. Conducting post-operative MR DTI can help correlate any damage to tracts and worsening neurological deficits, which can have therapeutic implications in future.

## Conclusions

This study has shown that DTI can potentially predict a higher grade of tumor in cases of astrocytoma based on the infiltration of WMTs. Oligodendrogliomas are more likely to cause infiltration of WMTs, irrespective of their grade. Patients with infiltration of WMTs on pre-operative scans do not necessarily develop post-operative worsening of neurological deficits. We recommend prospective studies with large sample sizes with control groups to validate our conclusion.
